# Detection of bacterial-reactive natural IgM antibodies in desert bighorn sheep populations

**DOI:** 10.1371/journal.pone.0180415

**Published:** 2017-06-29

**Authors:** Brian S. Dugovich, Melanie J. Peel, Amy L. Palmer, Ryszard A. Zielke, Aleksandra E. Sikora, Brianna R. Beechler, Anna E. Jolles, Clinton W. Epps, Brian P. Dolan

**Affiliations:** 1 Department of Biomedical Sciences, College of Veterinary Medicine, Oregon State University, Corvallis, Oregon, United States of America; 2 Department of Pharmaceutical Sciences, College of Pharmacy, Oregon State University, Corvallis, Oregon, United States of America; 3 Department of Fisheries and Wildlife, College of Agricultural Sciences, Oregon State University, Corvallis, Oregon, United States of America; International Nutrition Inc, UNITED STATES

## Abstract

Ecoimmunology is a burgeoning field of ecology which studies immune responses in wildlife by utilizing general immune assays such as the detection of natural antibody. Unlike adaptive antibodies, natural antibodies are important in innate immune responses and often recognized conserved epitopes present in pathogens. Here, we describe a procedure for measuring natural antibodies reactive to bacterial antigens that may be applicable to a variety of organisms. IgM from desert bighorn sheep plasma samples was tested for reactivity to outer membrane proteins from *Vibrio coralliilyticus*, a marine bacterium to which sheep would have not been exposed. Immunoblotting demonstrated bighorn sheep IgM could bind to a variety of bacterial cell envelope proteins while ELISA analysis allowed for rapid determination of natural antibody levels in hundreds of individual animals. Natural antibody levels were correlated with the ability of plasma to kill laboratory strains of *E*. *coli* bacteria. Finally, we demonstrate that natural antibody levels varied in two distinct populations of desert bighorn sheep. These data demonstrate a novel and specific measure of natural antibody function and show that this varies in ecologically relevant ways.

## Introduction

Ecoimmunology seeks to explain variation in immunity within and among hosts by examining immunity in the context of the host’s ecology and life history. Because immune responses involve a complex network of protein and cellular signals and effectors, a central conundrum in the field of ecoimmunology is “what to measure” [1]. Moreover, eco-immunological assays must reliably quantify immune responses in non-model species, including wildlife. In designing eco-immunological assays, the challenge is thus two-fold: First, to identify immune components that are relevant, by demonstrating that their level is indicative of functional immune responses, and second, to design robust assays that can capture variability in the immune component among hosts and populations of non-model animal species.

Natural antibodies (nAbs) are one innate immune component that is frequently measured in ecoimmunological studies. NAbs are present early in an animal’s development and can provide innate-like immune protection. NAbs are generated by a subclass of B cells known as B-1 B cells, which are distinct from typical B cells and tend to localize to the peritoneal cavity, bone marrow, and marginal zone of the spleen [2]. They are distinct from adaptive antibodies, which are highly specific to particular pathogens to which the host has previously been exposed to. Natural antibodies are mostly of the IgM subclass of antibodies and tend to have more gene rearrangements lacking N-nucleotide additions than adaptive antibodies [3, 4]. While they may be considered cross-reactive, nAbs are not non-specific, rather, nAbs recognize conserved antigenic epitopes present on multiple microbial agents [5].

NAbs have multiple functions [6]. NAbs are known to protect against bacterial infections [7–10] presumably by binding to conserved bacterial antigenic determinants. They can also provide a first line defense against viral infections [9, 11–14]. In addition to their innate immune functions, nAbs can also bind to self-antigens and are necessary for clearing apoptotic bodies [15–17], which is important in limiting the development of autoimmune diseases [18, 19]. Therefore nAb production is necessary for both protection and maintain physiological homeostasis [20].

Because they are present from birth and do not require vaccination or exposure to particular antigens to be generated, nAbs can be studied in a variety of wild vertebrate animals. One common technique relies on the ability of natural antibodies in serum samples to agglutinate red blood cells from a different species [21] by binding to particular blood group glycans. However, hemagglutination assays can be hampered by factors in serum other than antibodies [22–24]. Other tests have been developed that examine antibody binding to a foreign protein to which study animals had not previously been exposed, such as keyhole limpet hemocyanin (KLH) [25–27] or hen egg ovalbumin [28]. While these methods provide an inexpensive and repeatable way for determining nAb levels in a variety of organisms, they do not directly address if nAbs bind to bacterial epitopes to provide initial immune protection from bacterial infections.

To address these limitations, we developed a novel method for detecting anti-bacterial nAb levels in desert bighorn sheep (*Ovis canadensis nelsoni*) that does not rely on assessing binding to allogenic blood group antigens or a single foreign protein. Bighorn sheep are an iconic wildlife species that is susceptible to several infectious diseases which historically and currently have devastated wild populations [29–31], including in the desert subspecies native to the southwestern United States and northern Mexico. We tested the ability of plasma-isolated desert bighorn sheep IgM to bind to cell envelope proteins of *Vibrio coralliilyticus*, a marine bacterial pathogen that infects corals and oysters [32, 33]. Because bighorn sheep have not been exposed to this bacterium, no acquired antibody will be present to cross-react with the bacterial targets. Measuring nAbs which react to the surface antigens of a foreign bacterium is more likely to provide ecologically relevant information regarding an animal’s ability to withstand novel infections.

Importantly, we validated our assay in two ways. First, we evaluated the assay’s ecological relevance, by assessing its ability to capture immunological variation among distinct desert bighorn sheep populations. Second, we tested the assay’s functional relevance by comparing nAb levels with bactericidal activity of desert bighorn sheep plasma. Bactericidal capacity of blood or plasma is a commonly used eco-immunological measure of innate immune function. Bacterial killing assays are easily interpreted, because they directly measure variation among animals in their ability to eliminate bacterial invaders from their blood. We find that our assay detects prominent differences in nAb levels between two geographically isolated populations of desert bighorn sheep; and that nAb levels established by our assay correlate with variation in plasma killing capacity among individual bighorn sheep.

## Materials and methods

### Plasma sample collection

Plasma was obtained from blood samples collected from wild bighorn sheep captured in 2014 for ongoing research on recent respiratory disease outbreaks in the Mojave Desert and Peninsular Range of California [34]. Blood was collected from animals immobilized without anesthetics or sedatives into heparinized blood collection tubes via aseptic technique. Within 4 hours of collection, blood was centrifuged at 5000g for 10 minutes. The plasma was pipetted off the top and stored in micro centrifuge tubes at -80 until processing. Domestic sheep plasma was donated from Oregon State University Veterinary Teaching Hospital. Protocols for bighorn sheep capture were approved by the National Park Service Institutional Animal Care and Use Committee (permit PWR_MOJA_Epps.Powers_DesertBighorn_2013.A3).

### *V*. *coralliilyticus* culture and cell envelope isolation

*V*. *coralliilyticus* strain RE22 [35] was provided by Claudia Hase (Oregon State University) and grown in 3% NaCl LB broth at 30°C. For isolation of cellular envelopes, 2 ml of an overnight culture of *V*. *coralliilyticus* was used to inoculate 1 L of media, and bacterial cells were grown for 4 hours until an optical density of ~0.5 was measured at 600 nm. Cells were pelleted by centrifugation for 20 minutes at 7500 RCF. After carefully decanting the supernatant, the cell pellet was frozen at -20°C. Cell envelope proteins were isolated following previously established methods based on French press cell lysis, followed by treatment with chaotropic reagent (sodium carbonate) and differential centrifugation [36–38]. In comparison to other sub-fractionation methods this protocol allows an enrichment of outer membrane proteins, yielding a sample compatible with mass spectrometry. Briefly, *V*. *corallyticus* cell pellets were re-suspended in pre-chilled 1 × phosphate buffered saline pH 7.5 (PBS), supplemented with EDTA-Free Protease Inhibitors Tablet (Thermo-Fisher), and lysed by passage through French press cell (Amco) at 12,000 psi. Cell debris was removed by centrifugation at 16,000 RCF. Protein concentration was measured using Bio-Rad Protein Assay, and 10 mg was mixed with 6 volumes of 0.1M sodium carbonate pH 11.0. Samples were incubated with mixing for 1 hour at 4°C, and membrane proteins were pelleted at 200,000 × g using Type 45 rotor (Beckman). The pellet containing cell envelope proteins was re-suspended in ice cold PBS containing 0.1% SDS. Protein concentration was determined using a Bradford Assay (Bio-Rad).

### SDS-PAGE and western blotting

Protein samples were analyzed by SDS-PAGE using precast 4–12% Bolt^®^ Gels, and blotted using the iBlot2^®^ dry transfer system (Thermo-Fisher). Ten μl of LDS sample buffer (Thermo-Fisher) was added to 30 μl of sample, and the mixture was boiled for 20 minutes. The gel was loaded with 25 μl of sample and run at 165 volts, until the dye fully exited the gel. Gels were then blotted onto nitrocellulose membranes using iBlot^®^ program 3. Membranes were blocked in Tri-buffered Saline containing 0.1% Tween-20 (TBS-T) with 5% dehydrated milk. For the analysis of IgM, plasma was diluted 1:10 in water containing 10mM DTT. Bighorn sheep IgM was then detected on blocked membranes using a rabbit polyclonal anti-sheep IgM antibody (Bio-Rad Cat# AHP950) diluted 1:2000 in 0.5% milk/TBS-T, followed by goat-rabbit polyclonal antibody coupled to IR dye 800 (LI-COR), diluted 1:10,000 in 0.5% milk/TBS-T. For the analysis of bighorn sheep IgM binding to *V*. *coralliilyticus* membrane proteins, protein solutions (approximately 0.7 mg/ml) were diluted 1:1 with water prior to mixing with LDS sample buffer and loading onto the gel. After blocking membranes, bighorn sheep plasma was diluted 1:100 in TBS-T containing 0.5% milk, and added to the membranes. Membranes were gently rocked overnight, and the following day washed with TBS-T for 5 minutes. Rabbit anti-sheep IgM (diluted 1:2000, in 0.5% milk/TBS-T) was added to the membranes and incubated for 1 hour with gentle rocking. Membranes were washed in TBS-T, and goat-rabbit polyclonal antibody coupled to IR dye 800 (1:10,000 in 0.5% milk/TBS-T) was applied to the membrane for 1 hour with gentle rocking. For all western blots, following the final incubation with detecting antibodies, membranes were washed with TBS-T for 5 minutes followed by water for 5 minutes. Antibody binding was then visualized using an Odyssey SA infrared detection system.

### IgM and nAb ELISA

Bighorn sheep IgM was quantified by ELISA using a kit from Life Diagnostics (Cat# IGM-12) following the manufacturer’s recommendations. Samples were analyzed in duplicate and converted to a concentration of IgM using standards included in the kit. To determine nAb binding, 96-well polystyrene were coated with 350 ng of *V*. *coralliilyticus* membrane protein in 100 μl of PBS overnight at room temperature. ELISA plates were washed twice and blocked with 300 μl of 1% bovine serum albumin in PBS for 1 hour. Plates were washed five times, and 50 μl of bighorn sheep plasma, diluted 1:2000 in PBS, was added to each well. To test specificity of the ELISA, selected plasma samples were diluted in *V*. *coralliilyticus* cellular envelope proteins prior to addition to coated and blocked ELISA plates. Plates were gently mixed on an orbital shaker for 1 hour at room temperature, to allow nAb to bind to bacterial proteins. ELISA plates were then washed five times, and 100 μl rabbit anti-sheep IgM antibody coupled to HRP (Bio-Rad Cat# AHP950P), diluted 1:5000 in PBS, was added. Following one hour incubation, ELISA plates were washed seven times and 50 μl of TMB substrate (Amresco) was added to each well. After ~ 10 minutes, the peroxidase reaction was stopped by adding 100 μl 0.18M sulfuric acid. The wash buffer for all ELISAs was PBS containing 0.05% Tween-20. Absorbance measurements at 450 nm for all ELISA analysis were obtained with an Epoch plate reader (Biotek).

### Bacterial killing assay (BKA) and growth analysis

Lyophilized ATCC 8739 *E*. *coli* (Microbiologics) cells were re-suspended at 1 pellet/10ml PBS and diluted 10-fold in PBS to an approximate concentration of 10^5^ cells/ml. Plate-grown *V*. *corralliilyticus* was inoculated using the BBL Prompt System (BD) and diluting the initial kit-included inoculation tube of sterile saline 10-fold in PBS. Bighorn sheep plasma samples were diluted 1:10 in PBS and 40 μl of plasma was mixed with 10 μl of diluted *E*.*coli* or μl diluted V. coralliilyticus in sterile 96-well plates and incubated on an orbital shaker (200 RPM) for 30 minutes at 37°C. In some experiments, diluted plasma was heat-inactivated by incubation at 56°C for 30 minutes prior to exposure with bacteria. At the end of the incubation, 100 μl of Tryptic Soy Broth was added to each well containing *E*. *coli* or 100 μl of 3% NaCl LB broth was added to wells containing *V*. *coralliilyticus* and the absorbance at 600 nm recorded. The plates were incubated at 37°C until the conclusion of the experiment. Starting at five hours after the start of the incubation period, the absorbance at 600 nm was recorded hourly. The experiment was concluded after 14 hours. Bacteria incubated without plasma served as a control. Absorbance was plotted as a function of time. Growth curves had a predicted lag time, followed by a linear increase in absorbance, most often followed by a plateau. Data points within the linear range of growth were used to plot a linear regression for each individual; all individuals had at least 3 data points within the growth curve. The time to 0.3 absorbance (the approximate middle of the growth curve) was calculated from the linear regression using the regression formula for each individual and the values were natural log-transformed. In some plasma samples, bacterial growth was not detected at all over the course of the 14 hour incubation. Such samples were assigned a value of 15 hours for statistical analysis. For the purposes of graphical representation, animals were classified as “absolute killing”, “intermediate killing” or “no killing”. Plasma samples which did not inhibit bacterial growth, as defined by having a time to 0.3 absorbance less than or equal to the average time calculated from four control growth curves, were classified as “no killing.” Plasma samples which delayed the growth of *E*. *coli*, as defined by having some growth, but a time to 0.3 absorbance greater than the control samples, were labeled “intermediate killing.” Samples that never exhibited growth were classified as “absolute killing”.

### Data analysis

All analyses were performed in Graphpad Prism, except for general linear mixed modeling which was done using R studio package, version 3.1 with the nlme package [39]. For ELISA results we used a non-parametric t-test with Bonferroni correction to compare the ELISA result from an individual with maximal average staining (Sample 1) to an individual with minimal staining (Sample 2), anti-IgM alone and a negative control (BSA) (Bonferroni alpha<0.017). We then evaluated the correlation between all samples for IgM and the *V*. *coralliilyticus* reactive IgM using a linear regression. We used a linear regression to compare the *V*. *coralliilyticus* reactive IgM and the BKA on an individual level and to compare bacterial growth between *E*. *coli* and *V*. *coralliilyticus*. Finally, we evaluated if the *V*. *coralliilyticus* reactive IgM and the BKA differed between the Mojave and Peninsular sheep samples using generalized linear mixed models (Gaussian distribution, log link) with the main effect of population and a random effect of animal sex. Animal sex was used as a random effect because not all populations contained even numbers of males and females. Separate models were performed for *V*. *coralliilyticus* reactive IgM (dependent variable: natural log concentration) and the BKA (dependent variable: natural log of time to 0.3 absorbance, which is approximately mid-log growth).

## Results

### Desert bighorn sheep nAb bind to cell envelope proteins of V. coralliilyticus

**Fig 1:**
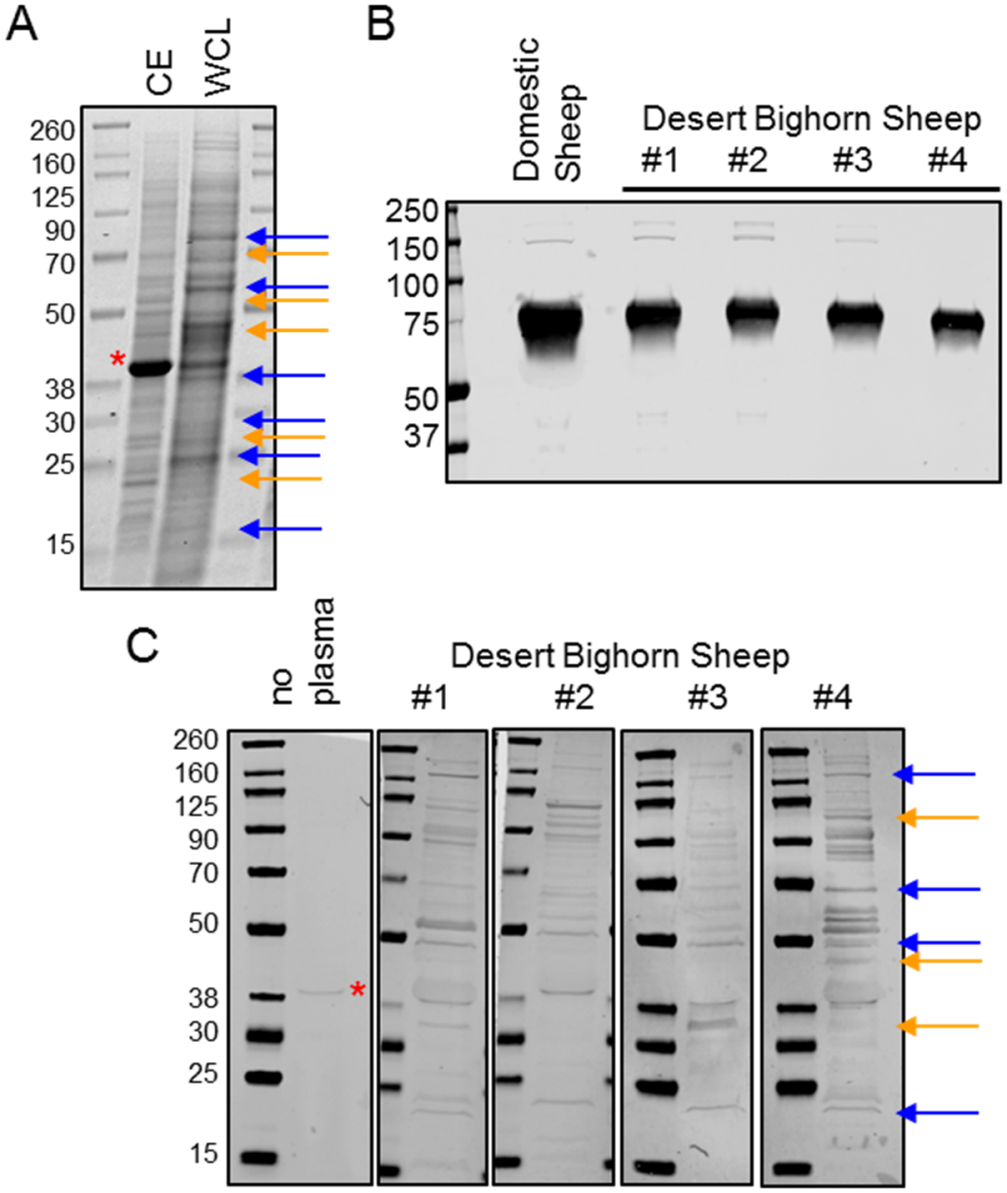
Desert bighorn sheep IgM binds to cell-surface proteins from the marine pathogen *V*. *coralliilyticus*. *A*. Cell envelopes (CE) and bacterial whole cell lysates (WCL) were separated by SDS-PAGE and visualized by staining with commassie blue dye. Molecular weight ladders flank the cell lysates and the weight of the protein (kDa) is denoted. Several distinct bands which are enriched in the CE fraction are marked orange arrows while bands absent from the membrane but present in the WCL are marked with a blue arrow. *B*. Plasma from domestic sheep or four randomly selected desert bighorn sheep were resolved by SDS-PAGE and transferred onto nitrocellulose. Rabbit anti-sheep IgM polyclonal antibodies were added to the membrane and detected with an anti-rabbit polyclonal antibodies coupled to a fluorescent dye. *C*. *V*. *coralliilyticus* cell envelope proteins were resolved by SDS-PAGE and blotted into a nitrocellulose membrane. Desert bighorn sheep plasma was added to the membranes, followed by rabbit-anti sheep IgM and anti-rabbit polyclonal antibodies coupled to a fluorescent dye. The image marked with an asterisk is a negative control, where the nitrocellulose membrane was not exposed to plasma. The asterisk also denotes a background band which is detected by the secondary antibodies. Several bands present in all animals tested are marked with blue arrows, whereas orange arrows denote bands which are only detectable in a subset of animals.

Some bacterial proteins, such as ribosomal proteins, are well-conserved across the prokaryotic kingdom, and adaptive antibodies generated against such antigens may cross react with proteins from *V*. *coralliilyticus*. To minimize this possibility, cell envelope proteins enriched in outer membrane protein fractions were isolated from *V*. *coralliilyticus*. As expected, the proteome of the cell-surface fraction was distinct from the whole cell lysate (Fig 1A), and many proteins present in the total cell lysate were absent in the membrane fraction. In addition to avoiding cross-reactivity with conserved intracellular proteins, the use of the bacterial-surface antigens ensured that we measured nAbs that bind to epitopes relevant for elimination of the pathogen, either by activating the classical complement system or enhancing opsonization by phagocytic cells.

To determine the extent of nAb binding, we utilized a commercially available rabbit anti-sheep IgM polyclonal antibody raised against domestic sheep IgM. When applied to bighorn plasma proteins separated by SDS-PAGE and blotted onto nitrocellulose, the anti-sheep IgM detected a band of approximately 75 kDa via western blot analysis, matching the predicted size of the bighorn sheep IgM heavy chain (Fig 1B) in four plasma samples. The heavy chain IgM band in bighorn sheep was the same size as domestic sheep (Fig 1B). Therefore, anti-sheep IgM polyclonal antibodies can be used to detect bighorn sheep IgM.

To determine if bighorn nAb could bind to bacterial proteins, we exposed *V*. *coralliilyticus* membrane proteins (separated by SDS-PAGE and blotted onto a nitrocellulose membrane) to bighorn sheep plasma, and subsequently detected bighorn IgM bound to bacterial proteins with rabbit anti-sheep IgM antibody. Plasma IgM from each animal showed some conserved and some unique protein bands (Fig 1C). The blue arrows in Fig 1C show bands which are detected in almost every animal tested, with varying levels of intensity. Orange arrows indicate bands which are only apparent in some individuals and not others. These data indicate that 1.) bighorn sheep IgM molecules can recognize cell-surface proteins from bacteria which do not infect bighorn sheep and 2.) slight variations in IgM binding, evident as variation in intensity of signal at bands recognized by all individuals or unique bacterial proteins recognized, exist in different individual animals.

#### Quantification of desert bighorn sheep nAbs using ELISA

**Fig 2:**
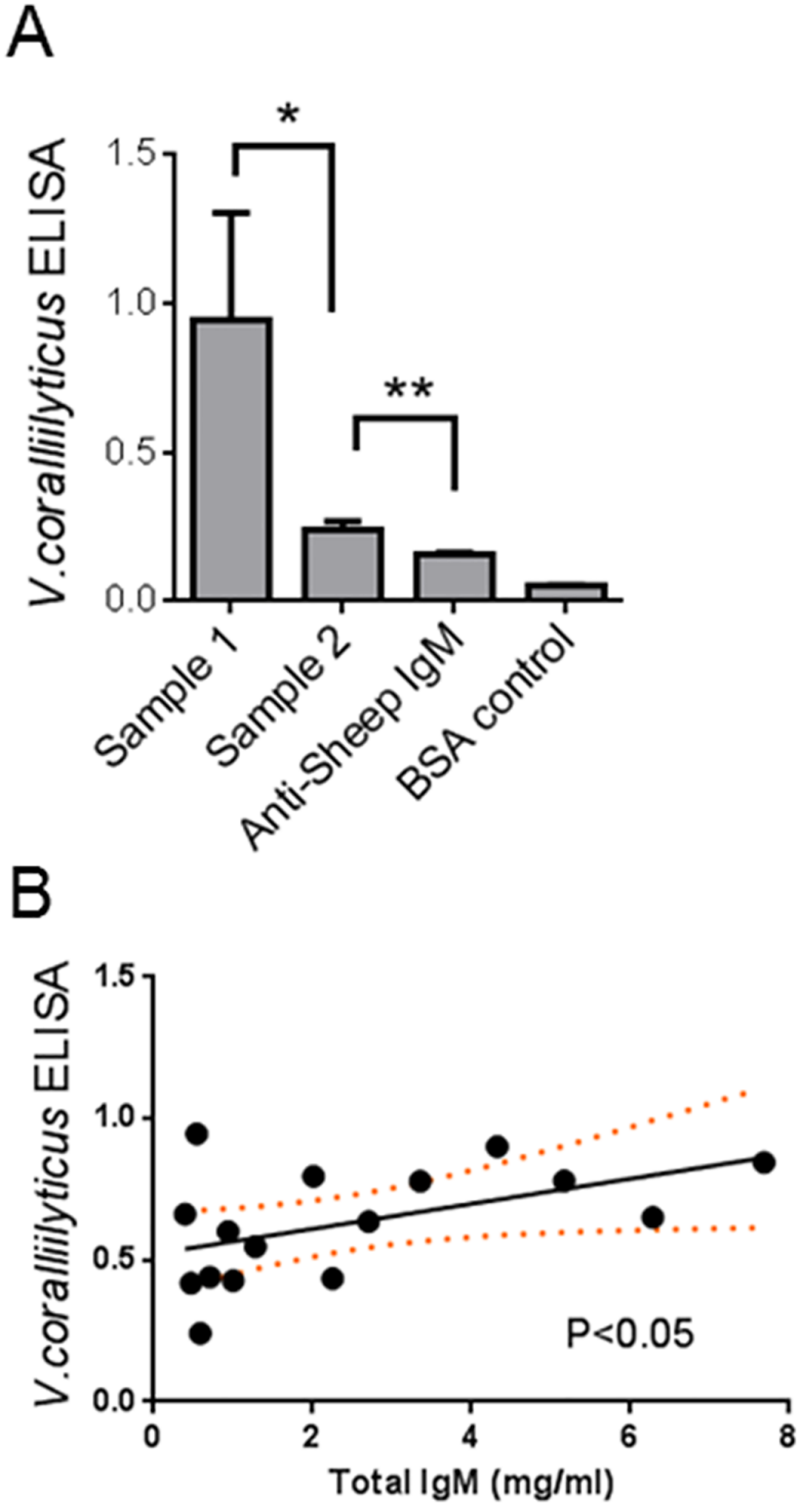
ELISA reliably detects desert bighorn sheep IgM binding to *V*. *coralliilyticus* cell envelope proteins. *A*. An ELISA for detecting desert bighorn sheep IgM binding to the membrane fractions of *V*. *coralliilyticus* was conducted with 17 random DBH sheep plasma samples. Samples were analyzed in triplicate and the individual with maximal average staining (Sample 1) and the minimal staining (Sample 2) are plotted with standard error bars. The background staining of anti-sheep IgM alone and BSA control wells is also shown (*, P<0.016, **, P<0.001). *B*. Average ELISA readings for the 17 plasma samples are plotted as a function of the total IgM determined by a separate ELISA analysis. The regression line is plotted showing 95% confidence intervals (orange dotted line) and the slope is statistically different from zero (P<0.05).

While immunoblotting provides visual confirmation of natural IgM binding to bacterial membrane proteins, the technique is notoriously difficult to quantify and not a cost-effective way to measure natural IgM levels in large populations of animals. An ELISA was therefore developed which utilized *V*. *coralliilyticus* cell envelope proteins as an antigenic target for IgM. A reliable signal could be detected and distinguished from all negative controls (Fig 2A). Depicted in Fig 2A are levels of bound IgM from two different animals representing the maximal and minimal signal detected in our initial assay, which included samples from 16 different individual bighorn sheep. The ELISA signal detected was specific, as pre-incubation of bighorn sheep plasma with *V*. *coralliilyticus* cell envelope proteins prior to addition to the ELISA plate reduced signal by 93% (n = 6, standard deviation = 12%). Variation in the ELISA signal from different animals might be due to differing overall levels of IgM in the plasma samples, rather than reflecting levels of nAb that bind specifically to *V*. *corelliilyticus* surface proteins. We therefore directly compared the *V*. *coralliilyticus* reactive IgM to total IgM determined using a commercially available kit (Fig 2B). Linear regression suggested that 75% of the variance in *V*. *corallillyticus* reactive IgM is unexplained by total plasma IgM (r^2^ = 0.25, p<0.05). Therefore, it is unlikely that natural IgM levels are directly related to total IgM levels in the plasma.

#### Assessing ecological and functional relevance: nAb levels differ between bighorn sheep populations, and correlate with bactericidal capacity

**Fig 3:**
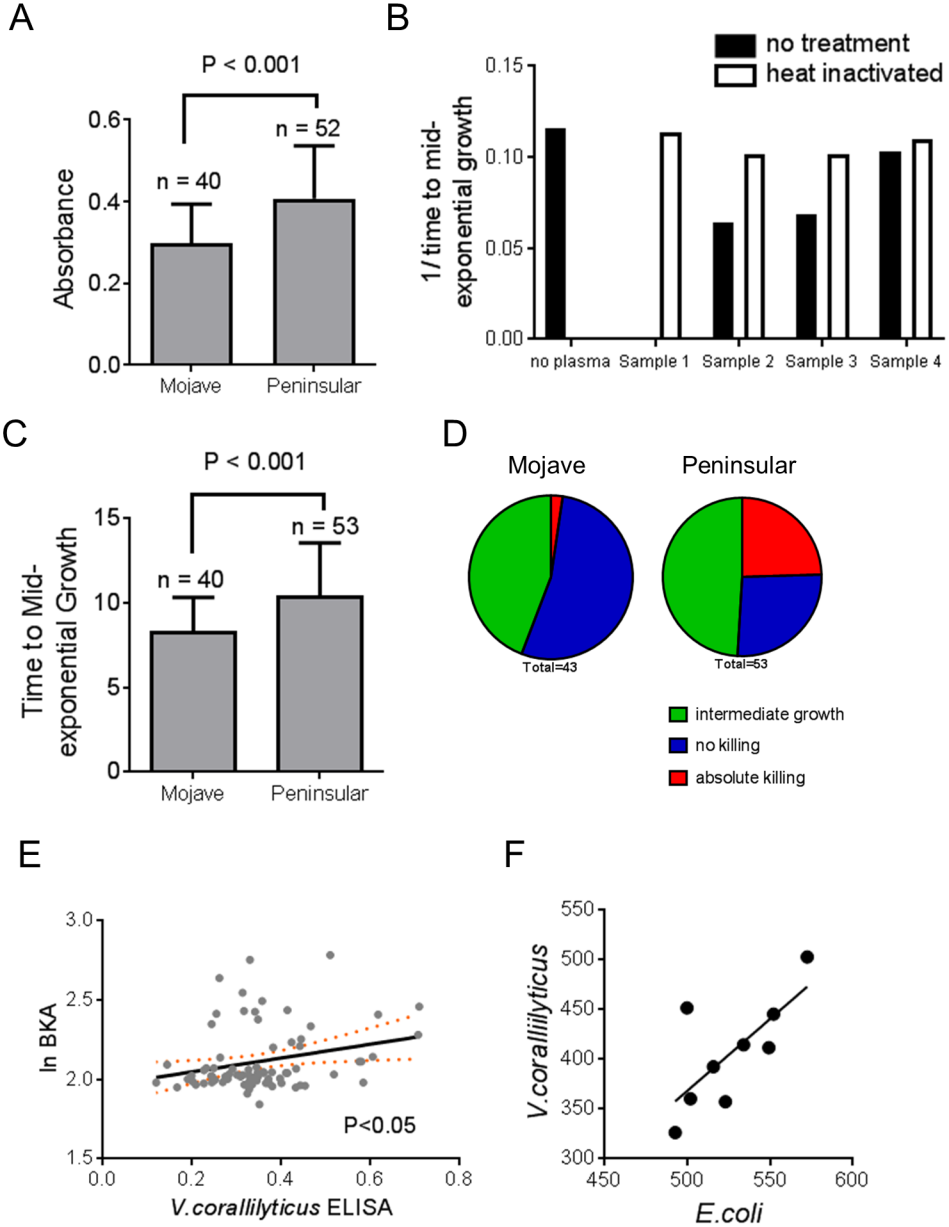
Natural IgM levels differ between geographically distinct desert bighorn sheep populations and correlate with bacteria killing capability of plasma. *A*. Average *V*. *coralliilyticus* ELISA values for each animal were averaged together by population location *B*. The ability of four bighorn sheep samples to kill laboratory grown *E*. *coli* for each population is depicted. Bacterial growth was monitored over a 14 hour period following exposure to plasma and the time to mid-exponential growth was calculated and technical replicates averaged. Here the inverse of time to mid-exponential growth is plotted. Plasma was either left untreated (black bars) or heat-inactivated prior to incubation with bacteria (white bars). *C*. The ability of plasma to prevent bacterial growth for each sample was determined and was then averaged for each population. *D*. Same as in (*C*) but each animal was classified as no-killing if bacteria growth was not delayed compared to untreated controls, intermediate killing if growth did occur, but was delayed compared to controls, or absolute killing if plasma prevented bacteria from growing at all. *E*. The natural log of the time to mid-exponential phase growth was compared to ELISA values for IgM binding to *V*. *corallilyticus* and the regression line is plotted with 95% confidence levels (orange dotted lines). The slope of the regression line is statistically different from zero (P<0.05). *F*. Nine bighorn sheep plasma samples were tested for the ability to kill both *E*. *coli* and *V*. *coralliilyticus* bacteria and the time to mid-exponential growth of each bacterial culture was compared. The slope of the regression line is statistically different from zero (P<0.05).

*V*. *coralliilyticus*-reactive nAb was determined by ELISA in 93 individual desert bighorn sheep from populations in two geographically-distinct regions (S1 Table). Natural antibody levels were higher in bighorn sheep sampled from California’s Peninsular Mountains when compared to bighorn sheep sampled from the Mojave Desert mountain ranges (Fig 3A, p<0.001), after accounting for sex as a random effect in a general linear mixed model.

Increased levels of nAb may enhance the ability of complement to kill bacteria, via the classical pathway. We therefore incubated plasma with *E*. *coli* bacteria and monitored bacterial growth over time. Some plasma samples were able to completely inhibit bacterial growth (Fig 3B Sample #1), while others delayed growth by several hours (Fig 3B Samples 2 and 3). There were also some samples which did not appreciably halt bacterial growth to any measurable extent (Fig 3B, Sample #4), at the dilutions used in this experiment. Heat-inactivation of plasma returned bacterial growth to control levels (Fig 3B), demonstrating that a heat-labile component, most likely complement proteins, is responsible for killing bacteria. We then compared the plasma killing of *E*. *coli* between sheep captured in the Peninsular and Mojave ranges. Bactericidal activity of Peninsular plasma samples was higher than samples taken from the Mojave (Fig 3C, p<0.001), after accounting for sex as a random effect in a general linear mixed model. In fact, over 50% of the plasma samples (23 out of 43 animals) collected from the Mojave range did not kill *E*. *coli* cells to a measurable extent, while only one plasma sample completely prevented bacterial growth (absolute killing) and the remaining animals (19 out of 43) showed intermediate killing (Fig 3D). In sharp contrast, ~25% of DBH plasma samples from the Peninsular range completely prevented bacterial growth while another 49% showed intermediate killing of bacteria, and the remaining 26% of individuals showed no inhibition of bacterial growth (Fig 3D). Because bactericidal activity of plasma is mediated by complement, we would hypothesize that plasma bacterial killing would be proportional to nAb levels, which could serve to activate the classical complement pathway. At the individual animal level we found that the bactericidal capacity of bighorn plasma showed a positive correlation with the amount of *V*. *coralliilyticus*-reactive nAb (Fig 3E, P<0.05). Finally, we determined that the ability of plasma to kill *E*. *coli* cells was highly correlated to the ability of plasma to kill *V*. *corallilyticus* cells (Fig 3F, P<0.05).

## Discussion

The field of ecoimmunology relies on a limited number of immune assays to document variation in immunity within and among wild animals. Experiments conducted with laboratory animals can utilize many different techniques to study natural antibody biology, including but not limited to, tracking specific B cell populations, analyzing germ-free or transgenic animals, expanding clonal B cells, and experimentally infecting animals. In wild animals, however, these techniques are not possible to implement. Two methods have commonly been used to measure nAb levels in a variety of wild animal systems: assessing the ability of nAbs to agglutinate red blood cells or to bind to a specific foreign protein, such as KHL or ovalbumin. It is widely held that nAbs are responsible for binding to foreign blood groups, however, not all nAbs may recognize foreign blood groups to the same extent that they recognize pathogenic bacteria signatures. In an study from Baxendale et al [40], natural antibody clones bearing non-mutated yet distinct heavy chains were able to bind to Pneumococcal capsular polysaccharides and ABO blood antigens with different affinities. Therefore, measuring erythrocyte agglutination may reflect the presence of natural antibodies with specificity for blood group antigens as opposed to bacterial products. KLH and ovalbumin are both glycoproteins and nAbs reactive to particular carbohydrate structures may bind to such targets. A vast heterogeneity of glycans is present on KLH [41], although ovalbumin tends to have fewer such structures [42]. However, without exact knowledge about which moieties are specifically recognized by nAbs, it is difficult to know the degree to which these glycans will be present on the foreign protein chosen for the assay. Furthermore, bacterial glycan structures are very diverse, using many monosaccharides with a variety of branch-structures [43], suggesting that bacterial-specific nAbs which react to specific polysaccharide epitopes may not react with KLH or ovalbumin. Additionally, nAbs can also react to lipid moieties, such as phosphocholine [7], which will not be detected by measuring antibody interactions with glycoproteins. We therefore chose to isolate bacterial cell envelopes, which would contain several components that could serve as potential targets for nAbs including bacterial-glycoproteins and lipids, as the antigen for nAb-binding in our assays.

Hunter et al. [44] demonstrated that western blot could be a powerful tool for discriminating between natural and acquired antibodies by examining the pattern of antibody staining to *Mycoplasma* antigens in naïve and infected desert tortoises. The binding of “naïve” bighorn sheep IgM to a pathogen also displays a particular pattern (Fig 1C) with several protein bands consistently appearing by western blot, though variations in intensity are apparent. Additionally, some animals appear to recognize unique bacterial proteins not recognized by other individuals. Whether these bands are the result of adaptive IgM cross-reacting to a specific bacterial protein, or the presence of a particular natural IgM antibody present in certain individuals, cannot be determined. It is also important to note that not all bacterial proteins present react with natural antibodies. The most prominent band in the membrane proteome (Fig 1A, marked with an asterisk) did not react with IgM antibody, nor did a group of low molecular weight proteins which migrated in the 15–25 kDa range on the gel. These data demonstrate that nAb IgM binding has specificity for particular bacterial epitopes and is not simply binding to proteins non-specifically.

Western blot data provided valuable information about the consistency with which particular proteins are recognized, but this is an impractical (and expensive) method for researchers who need to test hundreds of samples. For large sample sizes important in ecological studies, ELISAs are far more effective. Our data demonstrate that IgM antibodies can react to *V*. *coralliilyticus* cell envelopes bound to an ELISA plate. Importantly, the ELISA signal for natural antibody binding is not entirely explained by the total amount of IgM, with 75% of the variance left unexplained after accounting for total IgM. This finding also supports the conclusion that natural antibody reactivity to bacterial proteins is specific.

The bactericidal activity of plasma is most-likely mediated by the complement proteins present in serum, as heat-inactivation of plasma removed the bactericidal components (Fig 3B), however activation of the complement cascade requires recognition of the bacteria by other proteins such as antibody (either IgM or IgG) or lectins to be bound to the surface of the bacteria. We therefore anticipated that bacterial killing would be related to natural antibody levels. As shown in Fig 3E, levels of natural antibody and bacterial killing by plasma are correlated. Despite being correlated, the goodness of fit is relatively low (R^2^ = 0.06), which suggests that alterations in natural antibody levels can explain some, but not all of the variation in bactericidal activity between individuals. Other factors such as glycosylation of antibodies, adaptive IgG antibodies that react with bacterial antigens, or even variations with the numerous complement proteins also likely contribute to the observed bactericidal capability of plasma.

The data presented here indicate that nAb levels differ between populations of bighorn sheep, and that these differences may manifest in the ability of individuals to kill potentially infectious bacteria. Why these populations differ in the innate immune parameters we measured here is unknown, but could be the subject of future study. One possible explanation could be the different environmental conditions in the bighorn sheep populations. Elevation, precipitation, and access to water are known to have impacts on bighorn sheep populations, and differ between the two regions assessed in this study [45]. Differences in forage quality among mountain ranges could account for differences in innate immune responses, as antibody responses are known to be influenced by food intake [46]. Moreover, the Peninsular and Mojave metapopulations are genetically distinct [47], and have at times been considered separate subspecies [48, 49]. Mojave Desert populations exhibit varying degrees of habitat fragmentation and habitat conditions, both of which influence neutral and adaptive genetic diversity [50–52]. Thus, genetic factors could be responsible for the variation in nAb levels between bighorn sheep in the two regions. Different disease exposures could also lead to alterations in nAb levels as IgM secreted from B1 B cells rapidly increases following exposure to bacteria [53]. The Peninsular and Mojave bighorn sheep populations are known to have different disease histories [54]; higher prevalence of diseases in the Peninsular regions could predispose individuals there to produce more nAbs. Combining our approach to measuring relevant anti-bacterial natural antibody levels in wild animals with epidemiologic histories, monitoring, or natural experiments could clarify these hypotheses in this and other systems.

In conclusion, we developed an assay for measuring natural antibodies reactive to bacterial antigens that may be applicable to a variety of organisms. Our assay detects nAb with affinity for a broad range of cell surface proteins from a marine pathogen, *Vibrio coralliilyticus*, to which terrestrial animals are unlikely to have been exposed. We validated our assay by demonstrating that it captures prominent differences in nAb levels among bighorn sheep populations, and that this variation is associated with differences in actual innate immune function. For this iconic wildlife species, our assay thus provides an ecologically and functionally relevant measure of nAbs with affinity for bacterial epitopes. We hope that this novel assay for pathogen-specific nAb will serve as a useful tool in studying immunity in this, and other non-model species.

## Supporting information

S1 Table Data from individual animals in this study including bighorn sheep range, sex, calculated bacterial killing activity (BKA), and ELISA data of nAb levels reactive to *V*. *corralliilyticus* cellular envelopes is included. (XLSX)
